# Prognostic indicators of 6-month mortality in elderly people with advanced dementia: A systematic review

**DOI:** 10.1177/0269216312465649

**Published:** 2013-05

**Authors:** Meghan A Brown, Elizabeth L Sampson, Louise Jones, Anna M Barron

**Affiliations:** Department of Neuroscience, College of Letters, Arts, and Sciences, University of Southern California, Los Angeles, CA, USA; Marie Curie Palliative Care Research Unit, University College Medical School, University College London, London, UK; Marie Curie Palliative Care Research Unit, University College Medical School, University College London, London, UK; Department of Biophysics and Life Sciences, Graduate School of Arts and Sciences, The University of Tokyo, Tokyo, Japan

**Keywords:** Dementia, quality of life, palliative care, terminal care, prognosis, hospices

## Abstract

**Background:**

For end-of-life dementia patients, palliative care offers a better quality of life than continued aggressive or burdensome medical interventions. To provide the best care options to dementia sufferers, validated, reliable, sensitive, and accurate prognostic tools to identify end-of-life dementia stages are necessary.

**Aim:**

To identify accurate prognosticators of mortality in elderly advanced dementia patients consistently reported in the literature.

**Design:**

Systematic literature review.

**Data sources:**

PubMed, Embase, and PsycINFO databases were searched up to September 2012. Reference lists of included studies were also searched. Inclusion criteria were studies measuring factors specifically related to 6-month outcome in patients diagnosed with dementia in any residential or health-care setting.

**Results:**

Seven studies met the inclusion criteria, five of which were set in the United States and two in Israel. Methodology and prognostic outcomes varied greatly between the studies. All but one study found that Functional Assessment Staging phase 7c, currently widely used to assess hospice admission eligibility in the United States, was not a reliable predictor of 6-month mortality. The most common prognostic variables identified related to nutrition/nourishment, or eating habits, followed by increased risk on dementia severity scales and comorbidities.

**Conclusions:**

Although the majority of studies agreed that the Functional Assessment Staging 7c criterion was not a reliable predictor of 6-month mortality, we found a lack of prognosticator concordance across the literature. Further studies are essential to identify reliable, sensitive, and specific prognosticators, which can be applied to the clinical setting and allow increased availability of palliative care to dementia patients.

## Introduction

Palliative care can alleviate suffering and provide high-quality end-of-life care to dementia patients. Yet, despite similar levels of palliative-care need, dementia patients are substantially less likely to be referred for palliative care and are prescribed fewer palliative-care medications than terminally ill cancer patients.^1^ At the end of life, dementia sufferers are frequently hospitalized^2,3^ and commonly experience burdensome, invasive medical interventions, including tube feeding, laboratory tests, and restraint,^1^ and suffer from poor pain management.^4,5^ Dementia patients are also unlikely to have spiritual needs assessed before death.^6^ Failure to recognize dementia as a terminal illness often impacts end-of-life care provided to dementia patients.^1^ Furthermore, communication difficulties and a lack of advance directives, such as do not resuscitate orders,^7^ lead to a poor understanding of the needs and wishes of dementia patients. Currently, end-of-life care is predominantly provided to dementia patients by care homes, or to a lesser extent by informal carers in their own home.^8^ Complex treatment and social-care needs of dementia may mean that traditional palliative-care settings, such as hospices, are not appropriate or adequately equipped to provide care needs to dementia patients. These challenges are further confounded by difficulty in predicting prognosis of the terminal phase of dementia.

Dementia is characterized by prolonged and progressive disability, complicated by aging-related care needs and a high rate of comorbidity, making it difficult to identify the terminal phase of the disease. In order to provide the best care options to dementia patients, including the appropriate level of palliation, it is essential to identify and develop validated, reliable, sensitive, and accurate prognostic tools that can be used to identify end-stage dementia and that allow for advance preparation and planning. In the current work, we conduct a systematic review of recent studies aimed at identifying accurate predictors of 6-month mortality in elderly patients with advanced dementia so as to identify consistent prognosticators of mortality.

## Methods

Preferred Reporting Items for Systematic Reviews and Meta-Analyses (PRISMA) guidelines were followed in the processing and reporting of all results.^9^

### Search strategy

The literature was reviewed according to the guidelines for systematic reviews of prognostic factors outlined in Altman et al.^10^ The studies were identified by searches in PubMed, Embase, and PsycINFO electronic databases up to September 2012. A three-part search term was established in PubMed based on headings from the Medical Subject Headings (MeSH) of the National Library of Medicine and recommendations from the Altman guidelines:^10^

*Search term part 1.* ((“Dementia, vascular” [MeSH] OR “Dementia, Multi-infarct” [MeSH] OR “Alzheimer Disease” [MeSH] OR “Lewy Body Disease” [MeSH]) OR “Tauopathies”) AND (“mortality” [Title/Abstract] OR “survival” [Title/Abstract] OR “prognos* (*sic.*)” [Title/Abstract]))
OR
*Search term part 2*. “Dementia/Mortality” [Majr] (*sic.*)
OR
*Search term part 3.* (“Dementia” [MeSH] AND “6-month mortality”)

Comparable searches were performed in Embase and PsycINFO. Finally, the references of identified studies were also searched for any further relevant citations not obtained from the electronic databases.

### Inclusion and exclusion criteria

Specific inclusion and exclusion criteria were established to identify studies dealing exclusively with prognosticators of 6-month mortality in patients with advanced dementia (Table 1). Our exposure was advanced dementia of an organic origin, and our outcome was death within 6 months according to the natural progression of dementia (iatrogenic and acute illnesses were excluded).

**Table 1.** Inclusion and exclusion criteria.

Inclusion criteria	Exclusion criteria
Published in English	Not published in English
Human subjects	Nonhuman subjects
Description of demographics included	
Description of setting included	
Set in any residential or health-care setting	Includes acute admission to hospital as exposure
Measures factors specifically related to 6-month outcome	Measures results outside of a 6-month outcome
Measures specific exposure of advanced dementia	
Diagnoses of “dementia” or “advanced dementia” adhere to any validated criterion (or criteria)	
Includes diagnoses of Alzheimer disease, vascular dementia, multi-infarct dementia, and/or tauopathies	Includes diagnoses of MCI or early-stage dementia, AIDS dementia, delirium, prion disease, Rett syndrome, amyotrophic lateral sclerosis, and/or schizophrenia
Results in quantitative findings	Results in qualitative findings

MCI: mild cognitive impairment.

### Data extraction

From the selected abstracts, corresponding studies were read in full and analyzed according to the criteria established in Altman et al.^10^ A standardized form with the Altman characteristics was created and utilized to extract relevant data in a consistent manner from each selected study. Selected studies (1) met all of the inclusion criteria, (2) had clearly defined methodologies, (3) had well-defined participant populations of approximately the same stage of disease,^10^ (4) had follow-up times of at least 6 months per patient, (5) used methods of statistical analysis appropriate to their study methodologies, (6) reported demographic and clinical characteristics of the cohort populations, and (7) reported prognosticators of 6-month mortality as outcome results.

## Results

**Figure 1.**
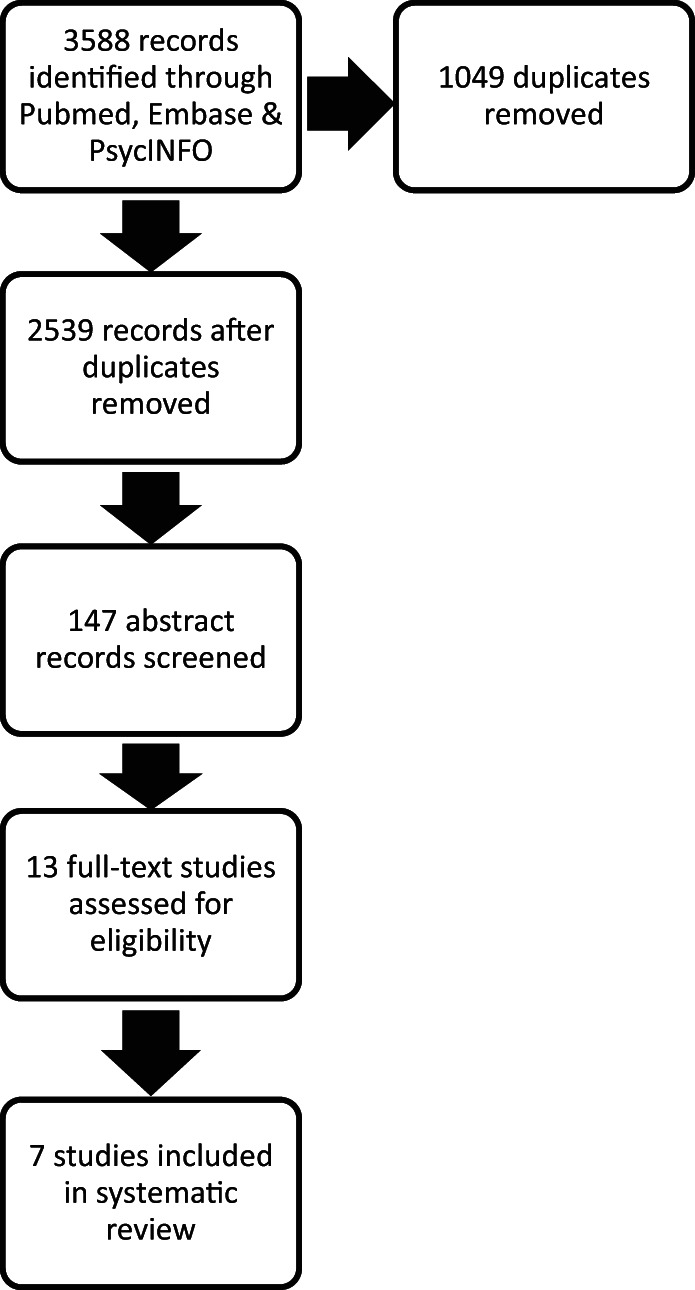
Flow chart of study identification for systematic review of prognosticators of 6-month mortality in advanced dementia.

The database searches yielded 2539 titles after duplicates were removed (Figure 1). Of these titles, 147 were chosen for review of their associated abstracts, from which 13 articles were chosen for consideration in the review. Upon reading, a further six articles were removed for failure to meet our inclusion criteria. After retrieval and exclusion of irrelevant articles, seven studies describing prognosticators of 6-month mortality in patients with advanced dementia were included in the review (Table 2).

**Table 2.** Characteristics of retrieved studies included in the systematic review. See Table 3 for explanation of scales utilized in diagnostic criteria.

Author	Methodology	Setting	*n*	Demographics		Diagnostic criteria	Follow-up duration	Statistical analysis
				Mean age (years)	Female (%)			
Aminoff^11^	Prospective cohort	Long-term care facility, Israel	103	51–96	56.3	*DSM-IV*, MMSE = 0, and FIM = 18	6 months	Fisher-exact test between 3 subgroups of MSSE scores and Kaplan–Meier analysis of survival curves with log rank and Breslow tests
Aminoff and Adunsky^12^	Prospective cohort	Long-term care facility, Israel	134	82.9	44.8	*DSM-IV*, primary physician, MMSE = 0, and FIM = 18	6 months	ANOVA and chi-square tests for associations between three subgroups of MSSE scores and univariate and multivariate Cox regressions between each criterion of MSSE
Luchins et al.^13^	Prospective cohort	Home and institutional hospice, United States	47	84	70	Primary physician	2-year study, minimum 6 months follow-up per patient	Initial univariate Cox regression models, secondary multivariate regressions and one-way ANOVA for NHO guidelines
Marsh et al.^14^	Descriptive methodological	Long-term care facilities, United States	112	82	75	Primary physician and score of 6+ on GDS	1-year study, 6 months follow-up per patient	Five logistical regression scales
Mitchell et al.^15^	Retrospective cohort	Nursing homes, United States	6799	83	66.8	Primary physician and cognitive performance score of 5 or 6 (score 5+ on MMSE)	6 months postadmission to nursing home	Cox proportional hazards model for unadjusted associations and stepwise Cox proportional hazards model for significant associations
Mitchell et al.^16^	Prospective cohort	Nursing homes, United States	606	65–>100	81.8	Primary physician and cognitive performance score of 6 (score 5+ on MMSE)	6 months	Area under the receiver operating characteristic score as a measure of discrimination of prognosticator. Proportional hazards regression model to estimate survival, observed mean, and predicted 6-month mortality compared with Hosmer–Lemeshow goodness of fit test
Schonwetter et al.^17^	Retrospective chart review	Institutional hospice, United States	165	83.5	62.7	Primary physician, KPS, and FAST	1-year study, 6 months follow-up per patient	Cox regression model to validate Medicare hospice guidelines, univariate and multivariate Cox proportional hazards analysis to identify prognostic factors, and multivariate prediction equation validated with second cohort

*DSM-IV: Diagnostic and Statistical Manual of Mental Disorders* (4th ed.); GDS: Global Deterioration Scale; KPS: Karnofsky Performance Scale; FAST: Functional Assessment Staging; MMSE: Mini-Mental State Examination; MSSE: Mini-Suffering State Examination; NHO: National Health Organization; ANOVA: analysis of variance; FIM: Functional Independence Measure.

### Study setting and participants

Of the seven studies analyzed, five were based in the United States^13–17^ and the remaining two in Israel.^11,12^ Two studies were set in hospices,^13,17^ two in nursing homes,^15,16^ and three in unspecified long-term care facilities.^11,12,14^ Luchins et al.^13^ included a cohort in a home hospice setting, while all remaining studies were set in institutionalized care facilities, whether hospice or other. Of those, at least one study was set in a for-profit-only center,^14^ and at least one other study was set in a not-for-profit-only center;^17^ the remaining studies failed to specify the profit status of their settings.^11–13,15,16^

The cohort sizes ranged from 47^13^ to 6799^15^ participants. All studies except Aminoff and Adunsky^12^ reported a majority of female participants, and the approximate average age of participants across the studies was 83 years (approximate range: 51 to over 100 years).

### Study methodologies

Several study methodologies were employed in the literature, including prospective studies^11–13,16^ and retrospective cohort studies,^15^ a retrospective chart review^17^ one descriptive methodological study.^14^

Each study had a follow-up period of 6 months per participant, though Schonwetter et al.,^17^ Luchins et al.,^13^ and Marsh et al.^14^ had a longer total study period. As criteria for acceptable diagnosis of dementia, five studies^13–17^ required clinical diagnoses from the primary physician, while the remaining two required a diagnosis based on the *Diagnostic and Statistical Manual of Mental Disorders* (4th ed.; *DSM-IV*) dementia criteria.^11,12^ Most studies also included at least one other rating scale for dementia severity, including the Functional Assessment Staging (FAST), Global Deterioration Scale (GDS), Mini-Mental State Examination (MMSE), and Mini-Suffering State Examination (MSSE) rating scales (Table 3).^11,12,14–17^

**Table 3.** Dementia diagnostic and rating scales.

Scale	Description	Relevance
ADEPT^18^	Continuous scale measuring 12 items	In Mitchell et al.:^16^ develops and validates the novel scale
	1. Nursing home stay <90 days	
	2. Age	
	3. Male	
	4. Shortness of breath	
	5. At least one pressure ulcer	
	6. ADLs score = 28	
	7. Bedfast	
	8. Insufficient oral intake	
	9. Bowel incontinence	
	10. Body mass index <18.5 kg/m^2^	
	11. Weight loss	
	12. Congestive heart failure	
	Total score range: 1.0–32.5, higher score indicates higher risk of death	
AHOPE^14^	Measures nine factors indicating symptom severity (score 1–4 per indicator)	In Marsh et al.:^14^ develops and validates the novel scale
	1. Level of consciousness	
	2. Eye contact	
	3. Speech	
	4. Muscle flexibility	
	5. Ambulation	
	6. Swallowing	
	7. Food intake	
	8. Fluid intake	
	9. Weight history	
	Score range: 9–36, higher score indicates increased severity of condition	
*DSM-IV*^19^	Establishes standardized diagnostic criteria for all mental disorders recognized by the American Psychiatric Association	In Aminoff and Adunsky^12^ and Aminoff.:^11^ standard diagnostic manual for clinical and community study of psychiatric disorders
FAST^15^	Total 16 ordinal phases, 1–7f, including 6d. Urinary incontinence occasionally or more frequently over the past weeks 6e. Bowel incontinence occasionally or more frequently in the past few weeks 7a. Speech limited to 1–5 words 7b. All intelligible vocabulary lost 7c. Nonambulatory 7d. Unable to sit independently 7e. Unable to smile 7f. Unable to hold head up All stages must be passed through sequentially	In Luchins et al.,^13^ Marsh et al.,^14^ Schonwetter et al.,^17^ Mitchell et al.,^15,16^ Aminoff and Adunsky,.^12^ and Aminoff.:^11^US National Hospice Organization recommends FAST stage 7c as criteria to enroll patients in hospice care, indicating prognosis of less than 6-month survival
FIM^20^	Seven-level test of 18 items to assess disability and medical rehabilitation outcomes. 1. Eating 2. Grooming 3. Bathing/Showering 4. Dressing upper body 5. Dressing lower body 6. Bladder management 7. Bowel management 8. Toileting 9. Transfers bed/chair/wheelchair 10. Toilet transfers 11. Bathtub/shower transfers 12. Locomotion: walking/wheelchair 13. Locomotion: stairs 14. Expression 15. Comprehension 16. Social interaction 17. Problem solving 18. Memory Each item scored from 1 to 7 based on level of dependence (1=complete dependence, 7=complete independence).	In Aminoff and Adunsky^12^ and Aminoff^11^
GDS^21^	Scale dividing the progression of degenerative dementia, from stage 1 (“no cognitive decline”) to stage 7 (“very severe cognitive decline”).	In Marsh et al.:^14^ basis of FAST
KPS^22^	Ordinal scale from 100 to 0, indicating functional status of patients with severe and terminal diseases; high scores indicate functional independence, score 0 indicates death	In Schonwetter et al.:^17^once considered a “gold standard” for staging progressive diseases
MDS /Mitchell Score^15^	Novel risk score developed by identifying 12 Minimum Data Set (MDS) factors that were indicated in 6-month mortality	In Mitchell et al.^15^ and Aminoff and Adonsky^12^
	1. ADLs score = 12	
	2. Male sex	
	3. Cancer	
	4. Congestive heart failure	
	5. O_2_ therapy in past 14 days	
	6. Shortness of breath	
	7. <25% of food eaten	
	8. Unstable medical condition	
	9. Bowel incontinence	
	10. Bedfast	
	11. Aged >83 yrs	
	12. Not awake most of the day	
	Max score 19	
MMSE^23^	11 questions divided into two parts: part 1 covers orientation, memory, attention, and verbal responses; part 2 looks at more complex tasks, including naming, following written commands, spontaneous writing, and copying a polygonal figure; max scare 30 (part 1: 21; part 2: 9; low scores indicate higher impairment)	In Marsh et al.,^14^ Aminoff and Adunsky,^12^ and Aminoff^11^
MSSE^24^	10-item scale consisting of symptoms of suffering and discomfort	In Aminoff and Adunsky^12^ and Aminoff^11^
	1. Not calm	
	2. Screams	
	3. Pain	
	4. Decubitus ulcers	
	5. Malnutrition	
	6. Eating disorders	
	7. Invasive actions	
	8. Unstable medical condition	
	9. Suffering according to medical opinion	
	10. Suffering according to family opinion	
	(0–10 scale); max score 10, with one point for each item present.	
	Low suffering = 0–3, intermediate = 4–6, severe = 7–10.	

ADL: activities of daily living; ADEPT: Advanced Dementia Prognostic Tool; *DSM-IV: Diagnostic and Statistical Manual of Mental Disorders* (4th ed.); GDS: Global Deterioration Scale; KPS: Karnofsky Performance Scale; FAST: Functional Assessment Staging; FIM: Functional Independence Measure; MMSE: Mini-Mental State Examination; MSSE: Mini-Suffering State Examination; AHOPE: Alzheimer’s-Hospice Placement Evaluation Scale; MDS score: Minimum Data Set Score; Mitchell Score: Mitchell Novel Risk Score.

With the exception of Aminoff^11^ and Mitchell et al.,^16^ all studies assessed the significance of prognostic indicators using regression models; of those, all but Marsh et al.^14^ based their regressions on the Cox model. Cox regression models were analyzed both at the univariate level and then at the multivariate level^12,13,17^ in a secondary stepwise function.^15^ Schonwetter et al.^17^ and Mitchell et al.^15,16^ also included validation cohorts; however, data from the validation cohorts were not considered in the systematic review. The specific hazard ratios (HRs) and confidence indices were only available for the results reported by Schonwetter et al.,^17^ Mitchell et al.,^15^ and Aminoff and Adunsky,^12^ while the remaining four articles did not report these standards in their findings.^13,14,16^

### Prognostic indicators of 6-month mortality

**Figure 2.**
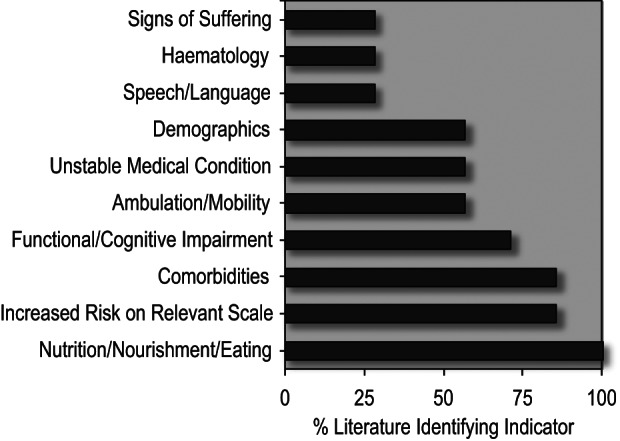
Prognostic indicators of 6-month mortality identified in examined literature.

Identified prognosticators of 6-month mortality varied greatly between studies (Figure 2 and Table 4). However, at least one factor relating to nutrition, nourishment, and/or eating habits was identified as a significant prognostic indicator in all studies, including decreased appetite,^13^ insufficient food intake,^15,16^ malnutrition,^11,12^ and weight loss.^14,16,17^ Anorexia was strongly and significantly associated with increased mortality within 6 months in multiple studies,^11,12,17^ and dry mouth, which impairs the ability to swallow, was found to be significantly associated with nearly doubled risk of mortality.^17^

**Table 4.** Identified prognostic indicators with strength of association with mortality (where given) of 6-month mortality in advanced dementia patients.

Category	Factors	Hazard ratio	95% confidence interval
Nutrition, nourishment, and eating	Decreased appetite^13^	Not given	Not given
	Anorexia^11,12,17^	2.22^17^	1.52–3.44^17^
	<25% food eaten^15,16^	1.5	1.4–1.7
	Dry mouth^17^	1.81^a^	1.23–2.67^a^
	Cachexia^17^	1.27^a^	1.03–1.55^a^
	Trouble swallowing, decreased fluid intake, and weight change as measured for AHOPE^14^	Not given	Not given
	General malnutrition^11,12^	Not given	Not given
Increased risk score on relevant scale	ADEPT^17^	Not given	Not given
	AHOPE 22+^14^	Not given	Not given
	FAST 7c^13^	Not given	Not given
	MDS/Mitchell 9+^15^	Not given	Not given
	MSSE 7+^11,12^	1.95^16^	1.17–3.25^16^
Comorbidities	Decubitus ulcers as measured for MSSE^16,17^/ADEPT^11^	Not given	Not given
	Cancer^12^	1.7	1.5–1.9
	Congestive heart failure^15,16^	1.6	1.4–1.7
	Incontinence^13,15,16^	1.5^5^	1.3–1.7^5^
	Generally, more comorbid conditions associated with decreased survival^13,15,17^	1.10^a,17^	0.99–1.21^a,17^
Functional/cognitive impairment	Decreased activities of daily living scores (28 or less)^13,15,16^	1.9^15^	1.7–2.1^15^
	Impairments associated with FAST 7c^13^	Not given	Not given
	Decreased KPS scores^17^	1.09	0.99–1.18
	Level of consciousness as measured for AHOPE^14^	Not given	Not given
	Not awake most of the day, as measured for MDS/Mitchell^15^	1.4	1.2–1.6
Ambulation/mobility	Ambulation as measured for AHOPE^14^	Not given	Not given
	Generally impaired mobility^13^	Not given	Not given
	Bedfast as measured for MDS/Mitchell/ADEPT^15,16^	1.5	1.3–1.7
Unstable medical condition	Oxygen therapy^15^	1.6	1.4–1.8
	Shortness of breath^15,16^	1.5^15^	1.3–1.9^15^
	Unstable medical condition as measured for MDS/Mitchell^15^	1.5	1.3–1.6
	Unstable medical condition as measured for MSSE^11,12^	Not given	Not given
Demographics	Aged 83+^15^	1.4	1.3–1.6
	Aged 87+^11^	Not given	Not given
	Married^17^	2.29	1.78–2.77
	Male^15,16^	1.9	1.7–2.1
Speech/language	Impaired as measured for FAST^13^	Not given	Not given
	Impaired as measured for AHOPE^14^	Not given	Not given
Hematology	Total hemoglobin^11,12^	1.13^12^	1.00–1.27^12^
	Total cholesterol^11^	0.995^12^	0.991–1.00^12^
	Total protein levels^11^	0.637^12^	0.497–0.817^12^
Signs of suffering	Not calm, screaming, pain, and suffering according to medical and family opinions, as measured for MSSE^11,12^	Not given	Not given

ADEPT: Advanced Dementia Prognostic Tool; FAST: Functional Assessment Staging; MMSE: Mini-Mental State Examination; MSSE: Mini-Suffering State Examination; AHOPE: Alzheimer’s-Hospice Placement Evaluation Scale; MDS: Mitchell Novel Risk Score.

Prognosticator category is listed in descending order of consensus between studies.

a Indicates results from univariate Cox regression models; all other results, where present, from Multivariate regressions.

Increased risk score on a dementia rating scale, such as the FAST, Mitchell Novel Risk Score (MDS/Mitchell score), and the Advanced Dementia Prognostic Tool (ADEPT) scales was a commonly identified risk factor in majority of the literature.^11–16^ Aminoff and Adunsky^11^ demonstrated that the MSSE scale might be particularly associated with increased mortality within 6 months (HR: 1.95, 95% confidence interval (CI): 1.17–3.25).

The presence of one or more comorbid conditions, including cancer and heart failure,^15^ was also identified as a significant prognosticator in majority of the literature.^11–13,15–17^ Furthermore, comorbidities were especially indicative of decline if more than one was present.^13,15,17^ Four of the seven studies also identified unstable medical condition^11,12,15,16^ and impaired mobility^13–16^ as significant prognosticators.

A majority of the literature also identified measures of functional or cognitive impairment as a significant prognostic indicator for 6-month mortality;^13–17^ the remaining two studies did not assess these measures.^11,12^ Mitchell et al.^15^ found that decreased activities of daily living scores were strongly and significantly associated with increased mortality. A definition of “not awake most of the day” was also identified as an associated risk factor.^15^

Other prognostic markers identified in the literature included speech and language deficits,^13,14^ hematological indices (hemoglobin, cholesterol, and total protein levels),^11,12^ and signs of suffering (screams and pain).^11,12^ Demographics were inconsistently identified as a prognosticator in the literature, with three studies finding age^11,15–17^ and two studies reporting sex^15,16^ to be significant prognosticators, while the other three studies^12–14^ found demographics to be insignificant in predicting mortality. Finally, the type of dementia was not found to be a significant indicator of 6-month mortality for end-stage patients in any of the studies.

## Discussion

### Main findings

For people with advanced dementia, palliative care may offer a better quality of life than continued aggressive or burdensome medical interventions.^3^ However, few studies have comprehensively addressed potential prognosticators for advanced dementia, with only seven studies identified in the current review fulfilling our inclusion criteria. Furthermore, the identified studies varied greatly in methodology and prognostic outcomes. The most common prognostic variables identified in the examined literature related to nutrition/nourishment, or eating habits, followed by increased risk on dementia severity scales and comorbidities.^11–17^ A majority of the studies also supported the inclusion of increased functional or cognitive impairment as a prognostic variable for 6-month mortality in dementia patient.^13–17^ Other possible prognosticators identified in over half of the studies included ambulation and mobility^13–16^ and unstable medical conditions.^11,12,15^ The potential clinical relevance of the prognosticators identified in the examined studies will be considered, particularly in the context of current guidelines used to enroll patients in palliative-care programs, which vary widely internationally.

### Methodological considerations

According to the guidelines for reviewing studies of prognostic markers established by Altman,^10^ the preferable study methodology in original research into prognostic variables is the inception cohort study. However, in the question of prognosticating mortality within 6 months in end-stage disease, this seems somewhat impractical, especially in the case of dementia. Early-stage dementia may very often go undiagnosed, making inception cohorts not only impractical but also nearly impossible to establish. In our case, a prospective cohort study would be the most preferable. In the literature presented in this review, four articles—Luchins et al.,^13^ Aminoff and Adunsky,^12^ Aminoff,^11^ and Mitchell et al.^16^—used a prospective cohort methodology. However, while a prospective cohort study design for in situ observation of the prognostic variables in the context of disease progression is preferable, it still poses problems for the investigation of prognosis in dementia. First, deciding when to enroll subjects into a prospective study may be difficult since staging dementia has proven inconsistent, making the requirement of a homogenous cohort difficult to fulfill. Second, with the exception of Mitchell et al.,^16^ most of the prospective cohort studies included in this review tended to have small participant populations.^11–13^ This may be due to the difficulty in gaining consent from a proxy, either due to inability to identify or contact a proxy,^16^ or in the case of identifying prognostic indicators of end of life, enrollment of patients indicates imminent grief to families and caretakers, and the associated psychosocial trauma may be unacceptable to these individuals. Retrospective analyses avoid this confounder, and chart and Minimum Data Set (MDS, a standard and mandatory list of clinical data on each patient in the United States) studies allow for a much wider candidate pool.^15^

Finally, interpretation of the importance of the identified candidate prognostic variables in the examined studies is difficult since the strength of association of prognostic indicators with mortality was not reported in many cases.^11,13,14^

### Current dementia mortality prognosticator guidelines

In the United States, where five of the studies included in this review were set, access to end-of-life care in the hospice setting is restricted to patients with a medically demonstrated prognosis of 6 months or less.^25^ Current National Hospice Palliative Care Organization (NHPCO) guidelines for assessing the eligibility of end-stage dementia patients for hospice care via Medicare benefits require: (1) sufficient dementia severity and (2) the occurrence of medical complications. Dementia severity is assessed using the FAST scale, and patients qualify for hospice admission when dementia severity surpasses stage 7c.^26^ In Israel, where the remaining two studies included in this review were set, legislation is also focused on 6-month prognosis, with terminally ill patients legally eligible for palliative care in the last 6 months of life under the ‘dying patient act’.^27^ In contrast, in the United Kingdom, the Gold Standards Framework (GSF) does not promote the development of care focused on time remaining but instead promotes planning and anticipation of worst-case scenarios in order to promote care driven by patient preferences.^28^ The GSF prognostic guidelines (which are widely used but have not been prospectively validated) aim to identify patients in the last 6–12 months of life, with the ABCD register classifying prognosis on a scale of years, months, weeks, or days, and this status is reviewed monthly.^28^

Our findings show that while most studies examined in the current review supported inclusion of a measure of dementia severity as a 6-month prognosticator of mortality,^11–16^ there was no consensus on the best scale to use. With the exception of the study by Luchins et al.,^13^ all the studies in this review found that the FAST 7c criterion currently used in the United States was not a reliable predictor of 6-month mortality.^11,12,14–17^ One limitation of the FAST scale is that it assumes an ordinal disease progression and therefore excludes all dementia patients whose disease progression is nonlinear.^13^ Particularly, this may exclude patients with comorbidities, who may skip stages as a result of secondary illnesses.^17^ Furthermore, FAST may not be valid for patients with non-Alzheimer dementia.^29^ Mitchell et al.^16^ found the ADEPT scale to perform moderately as a prognosticator for 6-month mortality, with high interrater reliability, good calibration, modest discrimination, and high sensitivity (>90%), although low specificity (30%). Compared to the ADEPT scale, Mitchell et al.^16^ found the US Medicare hospice eligibility guidelines to have poor discrimination. Aminoff^11^ found the MSSE scale to be particularly associated with increased mortality within 6 months, and previous studies have reported MMSE to have high specificity, but low sensitivity, especially with non-Alzheimer dementias.^30^

The findings of our systematic review also support the inclusion of a nutritional prognosticator into current guidelines. Anorexia was the most commonly cited prognosticator in the nutrition, nourishment, and eating category;^11,12,17^ however, anorexia may actually be a general risk factor for mortality, not specific to advanced dementia. Although the FAST scale used in current NHPCO prognosticator guidelines includes assessment of weight loss, which indirectly considers nutritional variables, the development of a separate prognostic scale for the assessment of nutritional variables in addition to a functional scale may increase prognosticator sensitivity. Since the methods used to assess nutrition varied widely between the studies—ranging between assessment of daily food consumption,^15,16^ weight loss,^17^ and malnutrition^12^—future studies should compare the prognostic value of these measures of nutrition to determine the most clinically relevant nutritional prognostic indicator.

Evidence for the presence of comorbidities as a prognostic factor was prevalent throughout the literature, with Mitchell et al.^15^ identifying cancer and congestive heart failure as particularly associated with 6-month mortality. However, because both these illnesses limit lifespan in general, and because as we age, we accrue more illnesses, it may be impossible to determine whether these comorbid conditions are independent predictors of mortality, or if and/or how they interact with dementia to further increase the risk of death.

Majority of the literature also supported the inclusion of increased functional or cognitive impairment as a prognostic variable for 6-month mortality.^13–17^ However, within the studies that assessed functional and cognitive impairment, there was a lack of concordance between methods measuring this variable, making any generalized analysis difficult, and clinical application impractical. However, in NHPCO guidelines, it is not assessed as an independent variable but as part of the FAST scale. As key symptoms of advanced and end-stage dementia, a consistent definition of functional and cognitive impairments and their specific significance needs to be determined by the field.

Some prognosticators that may prove useful, such as speech and language deficiencies, hematological indices, and signs of suffering, were not consistently evaluated throughout the literature. Therefore, it is difficult to determine their usefulness as potential prognosticators. Future studies should consider including these measures to conclusively evaluate their prognostic value.

### Sensitivity and specificity of candidate prognosticators

It is clinically important that prognosticators are sensitive, that is, they accurately identify mortality risk, yet specific to dementia. Previous systematic reviews on this topic have not considered dementia exclusively,^31,32^ thereby potentially reducing specificity of their findings, or have not considered studies examining mortality up to 24 months,^33^ which may reduce sensitivity of their findings. Yet, the prognosticators identified in the literature of the current review may still lack specificity to dementia diagnoses. Indeed, the most commonly identified prognosticators, including poor nutrition and comorbidities, are indicative of the risk of death in old age from a number of causes and correlate with all general nursing home mortality risks.^15^

Perhaps a reasonable future investigation would observe similar and different risk factors for end of life in advanced dementia versus other advanced terminal illnesses, so as to isolate the dementia-specific factors. Mitchell et al.^15^ have begun this and have identified the risk indices of time spent awake and need for oxygen therapy as specific to advanced dementia patients.

### Generalization of identified prognosticators

When establishing prognostic criteria that affect the level of care universally available to patients, special consideration needs to be taken to ensure that findings are translatable to all groups. All the studies included in this review were set in institutionalized care, whether hospice or other (with the exception of Luchins et al.,^13^ who included one cohort set in home hospice care). None of the studies examined cohorts based in community settings. While focusing on institutionalized care is practical in the context of assessing governmental standards that are only applied to medical and long-term care institutions, researchers must not ignore the reality of patients who continue to live in their own homes in the community, which may be very different from that in the medical or institutional care settings. The progression of disease may differ in a community setting, and palliative care may begin earlier and last longer than in the medical setting. Furthermore, the literature studied here did not include studies from the United Kingdom or Canada, so we were unable to compare findings across health-care systems (private vs universal).

Additionally, the demographic characteristics of the studies indicate that these study cohorts may not be fully representative of all dementia patients. In those studies that included ethnicity as a measure, the majority of each cohort was White.^13–15^ Furthermore, in all studies except the one by Aminoff and Adunsky,^12^ the majority of each cohort was female. Whether the findings of these studies can be translated to groups of different gender and ethnicity remains to be determined. In the case of the Mitchell et al.^15^ study, a follow-up report by Van der Steen et al.^34^ found that the MDS/Mitchell scale can, in fact, be generalized to populations outside the United States (specifically demonstrated in the Netherlands), as well as to populations not recently admitted to nursing homes (the original Mitchell et al.^15^ study specifically examined newly admitted nursing home populations). However, this study still investigates the MDS/Mitchell scale only in the context of a Western, majority White population. More studies of this nature including more diverse settings are needed to testify to the applicability of likely prognosticators before they are codified and applied to the general population.

### Clinical need of consistent and reliable dementia definition

A consistent definition of “advanced dementia” is needed that can be applied in a standardized manner across the literature or in clinical practice. In general, the criteria implemented in the examined studies lacked methodological uniformity between studies and in some cases, internally within the studies. Only Aminoff and Adunsky^12^ and Aminoff^11^ specified a particular diagnostic criterion that was applied equivalently to each participant (the *DSM-IV* dementia criteria). The criteria employed by Marsh et al.^14^ for determining advanced dementia (a score of 6 on the GDS) were perhaps not exclusive enough to identify patients with the most advanced diagnoses. The most critical element of a sound prognostic methodology is the inclusion of participants all at or about the same stage of disease,^10^ therefore, it is essential that such diagnostic criteria for advanced dementia are developed that can be applied both in research and clinically.

## Conclusions: clinical and practical implications

Future studies should address methodological limitations through inclusion of prospective cohorts where possible, and should be based in a wider range of settings, including community-, health-, and social-care settings. The demographics should be controlled to include as much of the affected population as possible, including patients of various racial, ethnic, and socioeconomic backgrounds; these demographics should be thoroughly reported in a reproducible manner. Likewise, future researchers must be diligent in including specific diagnostic criteria that were equivalently applied to each participant to again ensure reproducibility and methodological quality. Finally, future studies should aim to compare and identify those prognosticators that are similar and different between advanced dementia and diseases of old age in general, so as to more definitively isolate the factors that specifically apply to patients suffering in the end stages of advanced dementia. Successfully identifying accurate prognosticators may lead to increased availability of palliative-care options to dementia patients and their families in whichever setting their care is delivered.
